# Genetic variants of *ADAMTS7* confer risk for ischaemic stroke in the Chinese population

**DOI:** 10.18632/aging.102211

**Published:** 2019-08-28

**Authors:** Linfa Chen, Weidong Hu, Shengnan Li, Shaoyu Yao, Mengxu Wang, Xinglan Chen, Shaofeng Chen, Fu Deng, Peiyi Zhu, Keshen Li, Wangtao Zhong, Bin Zhao, Guoda Ma, You Li

**Affiliations:** 1 Guangdong Key Laboratory of Age-Related Cardiac and Cerebral Diseases, Affiliated Hospital of Guangdong Medical University, Zhanjiang 524001, China; 2 Department of Neurology, Affiliated Hospital of Guangdong Medical University, Zhanjiang 524001, China; 3 Institute of Neurology, Affiliated Hospital of Guangdong Medical University, Zhanjiang 524001, Guangdong, China; 4 Department of Nursing, Affiliated Hospital of Guangdong Medical University, Zhanjiang 524001, China

**Keywords:** ADAMTS7, ischaemic stroke, variant, CIMT, risk

## Abstract

Large-scale genome-wide association analyses show an association between *ADAMTS7* variations and coronary risk. However, the link between *ADAMTS7* variability and ischaemic stroke (IS) has yet to be determined. This study evaluated *ADAMTS7* variants with respect to the risk of IS. Genetic association analyses were performed in two independent case-control cohorts with 1279 patients with IS and 1268 age-matched healthy controls. Four variant genotypes of the *ADAMTS7* gene were identified using the Multiplex SNaPshot assay. The rs3825807, rs11634042, and rs7173743 variants of *ADAMTS7* were related to lower IS risk in both initial and replication cohort. The G-T-T-C and G-T-C-C haplotypes are significantly less prevalent in the IS group than in the control group. Further stratification according to IS subtypes indicated that carriers with the variant alleles of the rs3825807, rs11634042 and rs7173743 variants of *ADAMTS7*conferred a lower risk of developing large-artery atherosclerosis stroke subtype. Also, the mutated rs3825807 G allele, as well as the mutated rs11634042 T allele of ADAMTS7, are linked to a significant reduction of ADAMTS7 in patients with IS. Our findings confirm the role of ADAMTS7 in the pathophysiology of IS, with potentially significant implications for the prevention, treatment, and development of novel therapies for IS.

## INTRODUCTION

Stroke is a leading cause of morbidity and mortality worldwide independent of socioeconomic conditions [1]. Among all subtypes of stroke, 85% of deaths by stroke are attributed to ischaemic stroke (IS). This disease is promoted by multiple factors, such as genetics, hypertension, tobacco smoking, and diabetes [2]. Most IS cases are attributed to atherosclerosis, which is characterized by chronic inflammation of the vascular wall.

The ADAMTS family includes 19 proteases with multiple domains, disintegrin, and metalloproteinase activity, all of which feature thrombospondin-like motifs. This family prominently intervenes in several pathophysiological phenomena, such as remodeling of the extracellular matrix (ECM), angiogenesis, hemostasis, organogenesis, arthritis, and cancer [3]. ADAMTS proteinases have been observed to promote atherosclerosis in recent research [3, 4].

ADAMTS7’s structure includes a signal peptide, prodomain, metalloproteinase domain, disintegrin-like domain, as well as numerous thrombospondin type I repeats (TSP1) interspersed by spacer domains [5]. Cleavage of the ADAMTS7 propeptide is required for the maturation of ADAMTS7 [6]. Thrombospondin-5 (TSP 5,alsoor COMP) is the most well-known substrate of ADAMTS7 [7]. COMP is typically found in the ECM in chondral tissue and vessel walls, as well as in atherosclerotic and restenotic lesions, delineating its probable involvement in the pathophysiology of ECM remodeling [7]. Additional studies have indicated that ADAMTS7 promotes migration of vascular smooth muscle cells (VSMC) and hyperplasia of the intima layer *via* degradation of the inhibitory matrix protein COMP [7]. Functional ADAMTS7 is capable of impairing endothelial repair by degrading thrombospondin-1, resulting in impairment of both growth and migration of endothelial cells [8]. Recent *in vivo* evidence indicates mice lacking ADAMTS7 display diminished neointimal thickening, likely caused by reduced VSMC migration [9]. Furthermore, ADAMTS7 is expressed in human plaques at all stages, and higher ADAMTS7 levels correlate with a vulnerable plaque phenotype and increased risk for postoperative cardiovascular events [10]. Based on these findings, we hypothesize ADAMTS7 participate in the pathogenesis of IS.

In large-scale genome-wide association (GWA) investigations, variants of the *ADAMTS7* gene have been related to an increased risk of coronary artery disease (CAD) [11–14]. The rs3825807 (A/G) variant—a CAD-associated single-nucleotide polymorphism (SNP)—recently, has become the center of attention, as it is associated with an exchange of serine for proline in the ADAMTS7 prodomain with decreased processing of this structure [15]. Accumulating evidence from functional research suggests the rs3825807 variant in the coding region of *ADAMTS7* is functionally essential for the maturation of ADAMTS7 and the migration of VSMC [4, 15].

The *ADAMTS7* rs3825807 A allele has been associated with potentiation of VSMC migration and neointimal thickening, as well as the promotion of atherosclerosis and plaque rupture [16]. A recent study performed by Chan *et al.* demonstrated that CAD patients carrying the *ADAMTS7* rs3825807 G allele display atherosclerotic plaques with more stability as assessed by histopathology, better angiographic results, and a lower risk of revascularization at follow-up [17]. However, the *ADAMTS7-*IS relationship requires further exploration. In light of the potential role of ADAMTS7 in IS pathophysiology, we aimed to elucidate whether *ADAMTS7* variations are significantly linked with IS risk and, if so, what is the impact of these variants on the expression of ADAMTS7.

## RESULTS

### Demographics

A total of 2547 participants were enrolled; 1279 IS patients and 1268 healthy controls (Table 1). In two cohorts of our study, smoking, diabetes, and hypertension were significantly more frequent in the IS group. IS patients also had higher homocysteine (HCY), and lower high-density lipoprotein (HDL) cholesterol levels in two cohorts. No significant differences were found regarding age, serum uric acid, low-density lipoprotein (LDL) cholesterol, and total cholesterol between groups in two cohorts. Significant gender and triglycerides level differences were observed between IS patients and control in the initial cohort, whereas no differences regarding gender and triglycerides level were found between groups in the replication cohort.

**Table 1:** Characteristics of ischemic stroke cases and controls.

**Variables**	**Initial cohort**			**Replication cohort**		
	**IS (n=615)**	**control (n=615)**	***P value***	**IS (n=664)**	**control (n=653)**	***P value***
Mean age (years)	62.47±9.89	61.53±8.91	0.11	68.3±9.52	67.8±8.86	0.53
Male/female	408/207	311/304	< 0.001	441/223	403/250	0.22
Smokers, n (%)	167 (27.2)	73 (11.9)	**< 0.001**	168 (25.3)	112 (17.2)	**< 0.001**
Hypertension, n (%)	466 (75.8)	199 (32.4)	**< 0.001**	425 (64.0)	154 (23.6)	**< 0.001**
Diabetes, n (%)	184 (29.9)	51 (8.3)	**< 0.001**	179 (27.0)	60 (9.2)	**< 0.001**
uric acid (mmol/L)	319.8±88.4	322.1±90.9	0.72	336.4±78.3	328.2±84.5	0.32
Total cholesterol (mmol/L)	5.07±1.18	5.06±0.99	0.94	5.14±1.23	5.08±1.05	0.58
Triglycerides (mmol/L)	1.62±1.09	1.42±0.97	**< 0.001**	1.54±0.98	1.55±1.24	0.51
HDL-cholesterol (mmol/L)	1.30±0.64	1.41±0.42	**< 0.001**	1.32±0.57	1.55±0.83	**< 0.001**
LDL-cholesterol (mmol/L)	3.07±1.03	3.01±0.97	0.42	3.21±0.98	3.18±0.95	0.36
HCY (mmol/L)	11.60±6.06	10.33±3.33	**< 0.001**	12.36±4.85	10.52±2.56	**< 0.001**

HDL-cholesterol: high-density lipoprotein cholesterol; LDL: low-density lipoprotein cholesterol; HCY: Homocysteine

Continuous data are presented as the mean ± SD, median (range) or n (%).

^a^P < 0.05 is indicated in bold font.

### *ADAMTS7* variants and IS risk

**Figure 1:**
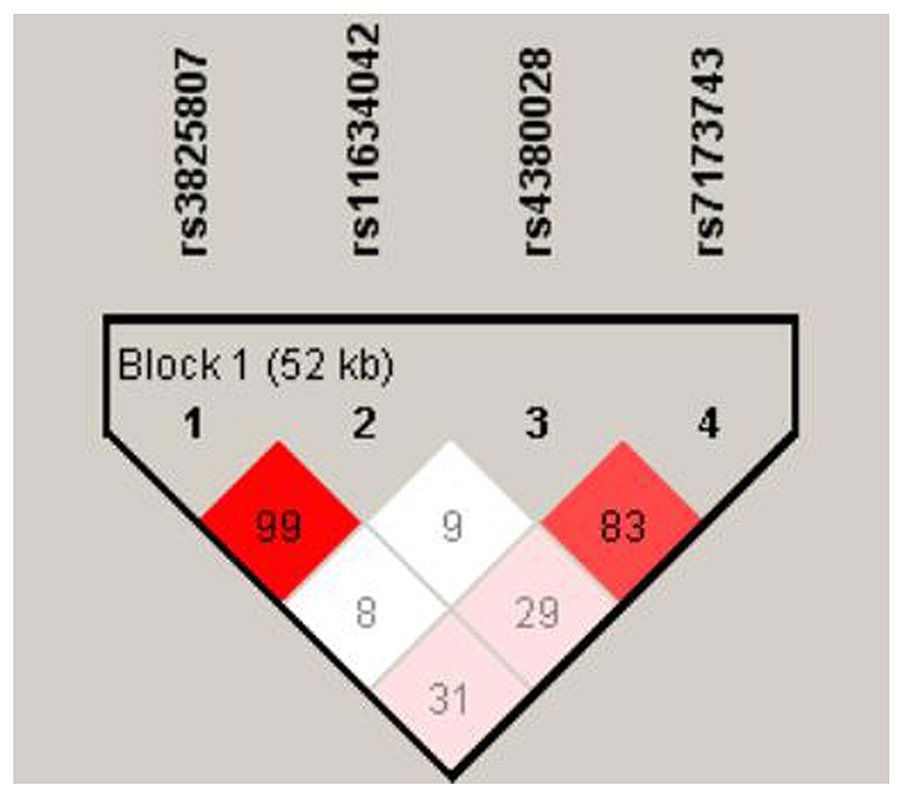
**The linkage pattern of polymorphisms in *ADAMTS7* gene.** The shade of the diamonds represents the pairwise r^2^ between the two SNPs as defined by the top left and top right sides of the diamond. Shading represents the magnitude of the pairwise r^2^, with red shades reflecting high r^2^ (>0.80) and white shades reflecting low r^2^.

Table 2 depicts the frequencies of the *ADAMTS7* genotype and allele variants. The assessed variants did not deviate from Hardy-Weinberg equilibrium (P > 0.05). Figure 1 shows the linkage pattern of polymorphism within the *ADAMTS7* gene. In the initial cohort, genotypic association analyses between IS patients and control revealed a statistical association between the rs3825807 and rs11634042 variants with IS risk (P = 0.0073 and P = 0.0071, respectively). This significant association was further confirmed in the replication cohort (P = 0.037 and P = 0.035, respectively). In a dominant model (AA vs. AG/GG and CC vs. CT/TT), significant differences in the frequency were observed for rs3825807 (P = 0.0040 in the initial cohort and P = 0.024 in the replication cohort) and rs11634042 (P = 0.0048 in the initial cohort and P = 0.026 in the replication cohort) in IS patients compared to the control group. In contrast, the recessive model did not identify significant differences between either the IS group or control group for the two variants (rs3825807 and rs11634042). However, in combined cohort, the recessive model showed a significant difference in IS patients compared with controls for rs3825807 and rs11634042 (P = 0.031 and P = 0.015, respectively). The frequencies of the variant G allele at rs3825807 (P = 0.0040 in the initial cohort and P = 0.024 in the replication cohort) and the variant T allele atrs11634042 (P = 0.0048 in the initial cohort and P = 0.026 in the replication cohort) were significantly greater in the IS group than in the control group. We did, however, find significant differences in the genotypic and allelic distributions of the rs7173743 variant of IS patients and the control group in two cohorts (P = 0.013 in the initial cohort and P = 0.025 in the replication cohort). Likewise, the dominant model (TT vs TC/CC) revealed a significant difference in rs7173743 frequency in IS patients in comparison with controls (P = 0.046 in the initial cohort and P = 0.047 in the replication cohort). In addition, the recessive model (TT/TC vs CC) also showed a significant difference in IS patients compared with controls (P = 0.013 in the initial cohort and P = 0.020 in the replication cohort). The frequency of the variant C allele at rs7173743 was significantly different in the IS group compared with the control group (P = 0.012 and P = 0.020 in the replication cohort). No statistical association was recognized between the rs4380028 variant and IS risk. Moreover, the same trend was observed for the heterozygous mutation after Bonferroni correction in combined cohort, which confirms the findings in both initial cohort and replication cohort. Since no significant heterogeneity in allele distribution between the two cohorts was detected, the two cohorts were combined for the subsequent analysis.

**Table 2:** Genotype and allele frequencies of *ADAMTS7* variants between IS patients and controls, and corresponding ORs for IS.

**Genotype & Allele**	**Initial cohort**				**Replication cohort**				**Combined cohort**	
	**IS patients (n=615)**	**Controls (n=615)**	**OR (95% CI)**	**P value^a^**	**IS patients (n=664)**	**Controls (n=653)**	**OR (95% CI)**	**P value^a^**	**OR (95% CI)**	**P value^a^**
**rs3825807**										
AA	462(75.1)	412(67.0)		**0.0073**	492(74.4)	443(67.8)		**0.037**		**2.4×10^-4^**
AG	140(22.8)	181(29.4)			158(23.8)	187(28.7)				
GG	13(2.1)	22(3.6)			14(1.8)	23(3.5)				
AA vs AG/GG	153(24.9)	203(33.0)	0.67(0.52-0.86)	**0.0040**	172 (25.9)	210 (32.2)	0.74(0.58-0.94)	**0.024**	0.71(0.59-0.84)	**1.3×10^-4^**
AA/AG vs GG	602(97.9)	593(96.4)	0.58(0.29-1.17)	**0.17**	650 (97.9)	630 (96.5)	0.59(0.30-1.16)	**0.11**	0.59(0.36-0.95)	**0.031**
A	1064(86.5)	1005(81.7)			1142(86.0)	1073(82.2)				
G	166(13.5)	225(18.3)	0.70(0.56-0.87)	**0.0040**	186 (14.0)	233 (17.8)	0.75(0.61-0.93)	**0.024**	0.72(0.62-0.84)	**1.2×10^-4^**
**rs11634042**										
CC	464(75.4)	415(67.5)		**0.0071**	496(74.7)	448(68.6)		**0.035**		**2.0×10^-4^**
CT	139(22.6)	178(28.9)			155(23.3)	182(27.9)				
TT	12(2.0)	22(3.6)			13(2.0)	23(3.5)				
CC vs CT/TT	151(24.6)	200(32.5)	0.68(0.53-0.87)	**0.0048**	168(25.3)	205(34.5)	0.75(0.59-0.95)	**0.026**	0.71(0.60-0.84)	**1.8×10^-4^**
CC/CT vs TT	603(98.0)	593(96.4)	0.54(0.26-1.09)	**0.12**	651(98.0)	630(96.5)	0.55(0.27-1.09)	**0.077**	0.54(0.33-0.89)	**0.015**
C	1067(86.7)	1008(82.0)			1147(86.4)	1078(82.5)				
T	163(13.3)	222(18.0)	0.69(0.56-0.86)	**0.0048**	181(13.6)	228(17.5)	0.75(0.60-0.92)	**0.026**	0.72(0.62-0.84)	**1.0×10^-4^**
**rs4380028**										
CC	211(34.3)	188(30.6)		**0.48**	233(35.1)	207(31.7)		0.27		0.14
CT	296(48.1)	309(50.2)			316(47.6)	314(48.1)				
TT	108(17.6)	118(19.2)			115(17.3)	132(20.2)				
CC vs CT/TT	404(65.7)	427(69.4)	0.84(0.66-1.07)	0.38	431(64.9)	446(68.3)	0.86(0.68-1.08)	0.25	0.85(0.72-1.00)	0.12
CC/CT vs TT	507(82.4)	497(80.8)	0.90(0.67-1.20)	0.51	549(82.7)	521(79.8)	0.83(0.63-1.09)	0.25	0.86(0.70-1.05)	0.14
C	718(58.4)	685(55.7)			782(58.9)	728(55.7)				
T	512(41.6)	545(44.3)	0.90(0.76-1.05)	0.38	546(41.1)	578(44.3)	0.88(0.75-1.03)	0.25	0.89(0.79-0.99)	0.12
										
**rs7173743**										
TT	166(27.0)	135(22.0)		**0.013**	186(28.0)	152(23.3)		**0.025**		**2.5×10^-4^**
TC	309(50.2)	297(48.3)			330(49.7)	315(48.2)				
CC	140(22.8)	183(29.7)			148(22.3)	186(28.5)				
TT vs TC/CC	449(73.0)	480(78.0)	0.76(0.59-0.99)	**0.046**	478(72.0)	501(76.7)	0.78(0.61-1.00)	**0.047**	0.77(0.64-0.92)	**0.0046**
TT/TC vs CC	475(77.2)	432(70.3)	0.70(0.54-0.90)	**0.013**	516(77.7)	467(71.5)	0.72(0.56-0.92)	**0.020**	0.71(0.59-0.85)	**2.5×10^-4^**
T	641(52.1)	567(46.0)			702(52.9)	619(47.4)				
C	589(47.9)	663(54.0)	0.79(0.67-0.92)	**0.012**	626(47.1)	687(52.6)	0.80(0.69-0.94)	**0.020**	0.79(0.71-0.89)	**1.7×10^-4^**

Data are presented as number (%).

^a^ adjusted for age, gender, smoking, hypertension, diabetes mellitus and hyperlipidaemia.

P value under 0.05 were indicated in bold font.

### Haplotype analysis

The G-T-T-C and G-T-C-C haplotypes (corresponding to the rs3825807–rs11634042–rs4380028–rs7173743 variants) were significantly less frequent in IS patients than control (P = 2.0×10^-6^ and P = 3.6×10^-4^, respectively). Adjustments for age, gender, smoking, hypertension, and diabetes mellitus, showed these two haplotypes to be linked with reduced IS risk in comparison to the most common A-C-C-T haplotype after the same adjustment (Table 3).

**Table 3:** The frequencies of haplotypes of *ADAMTS7* gene in combined cohort.

**Haplotypes**	**Case (freq%)**	**Control (freq%)**	**P value^a^**	**OR (95% CI)**
*ADAMTS7* (rs3825807, rs11634042, rs4380028, rs7173743)				
A-C-C-T	1093(42.8)	974(38.4)		1.00(reference)
A-C-T-C	815(31.9)	811(32.0)	0.11	0.90(0.79-1.03)
A-C-C-C	176(6.9)	188(7.4)	0.11	0.82(0.66-1.03)
G-T-T-C	112(4.4)	188(7.4)	**2.0×10^-6^**	0.57(0.45-0.73)
G-T-C-C	99(3.9)	147(5.8)	**3.6×10^-4^**	0.50(0.38-0.66)

Adjusted for age, gender, smoking, hypertension, diabetes mellitus and hyperlipidaemia.

All those frequency<0.05 will be ignored in analysis.

^a^ False discovery rate-adjusted P value for multiple hypotheses testing using the Benjamini-Hochberg method.

P value under 0.05 was indicated in bold font.

### Association between *ADAMTS7* variants and demographic characteristics

Tables 4–6 show the relationships between *ADAMTS7* variants and key demographic characteristics. After stratification by age, diabetes, and hypertension, the G allele at rs3825807 and the T allele at rs11634042 were associated with lower IS risk in patients under 70 years of age (P = 0.0015 and P = 0.0012, respectively), women (P = 0.0015 and P = 0.0012, respectively), nondiabetic patients (P = 0.0015 and P = 0.0012, respectively) and non-hypertensive patients (P = 0.0015 and P = 0.0013, respectively) (Tables 4, 5). Interestingly, stratification by age, gender, diabetes, and hypertension also attributed decreased risk to the C allele at rs7173743 in patients under 70 years of age (P = 0.0016), female (P = 1.4×10^-5^), nondiabetic patients (P = 4.0×10^-5^) and non-hypertensive patients (P = 0.0034) (Table 6).

**Table 4:** A comparison between the baseline characteristics of the *ADAMTS7* rs3825807 genotypes and alleles in combined IS patient and control groups.

**Characteristics**	**IS patient group**					**Control group**					***P_G_^a^ value***	***P_A_^a^ value***
	**Genotype n (%)**			**Allele n (%)**		**Genotype n (%)**			**Allele n (%)**			
	**AA**	**AG**	**GG**	**A**	**G**	**AA**	**AG**	**GG**	**A**	**G**		
Age												
≥70	389(75.4)	117(22.7)	10(1.9)	895(86.7)	137(13.3)	265(69.6)	104(27.3)	12(3.1)	634(83.2)	128(16.8)	0.15	0.057
<70	565(75.0)	181(22.8)	17(2.2)	1311(85.9)	215(14.1)	590(66.5)	264(29.8)	33(3.7)	1444(81.4)	330(18.6)	**0.0067**	**0.0015**
Gender												
Male	635(74.8)	199(23.4)	15(1.8)	1469(86.5)	229(13.5)	500(70.0)	198(27.7)	16(2.3)	1198(83.9)	230 (16.1)	0.15	0.057
Female	319(74.2)	99(23.0)	12(2.8)	737(85.7)	123(14.3)	355(64.1)	170(30.7)	29(5.2)	880(79.4)	228(20.6)	**0.022**	**0.0015**
Diabetes												
Yes	284(78.2)	71(19.6)	8(2.2)	639(88.0)	87(12.0)	82(73.9)	28(25.2)	1(0.9)	192(86.5)	30(13.5)	0.37	0.56
No	670(73.1)	227(24.8)	19(2.1)	1567(85.5)	265(14.5)	773(66.8)	340(29.4)	44(3.8)	1886(81.5)	428(18.5)	**0.0067**	**0.0015**
Hypertension												
Yes	662(74.3)	209(23.5)	20(2.2)	1533(86.0)	249(14.0)	247(70.0)	95(26.9)	11(3.1)	589(83.4)	117(16.6)	0.29	0.11
No	292(75.3)	89(22.9)	7(1.8)	673(86.7)	103(13.3)	608(66.5)	273(29.8)	34(3.7)	1489(81.4)	341(18.6)	**0.010**	**0.0015**

P_G_: P value of the difference in alleles between the case and control groups; P_A_: P value of the difference in genotype between the case and control groups

^*a*^ adjusted for age, gender, smoking, hypertension, diabetes mellitus and hyperlipidaemia.

P < 0.05 is indicated in bold font.

**Table 5:** A comparison between the baseline characteristics of the *ADAMTS7* rs11634042 genotypes and alleles in combined IS patient and control groups.

**Characteristics**	**IS patient group**					**Control group**					***P_G_^a^ value***	***P_A_^a^ value***
	**Genotype n (%)**			**Allele n (%)**		**Genotype n (%)**			**Allele n (%)**			
	**CC**	**CT**	**TT**	**C**	**T**	**CC**	**CT**	**TT**	**C**	**T**		
Age												
≥70	391(75.8)	115(22.3)	10(1.9)	897(86.9)	135(13.1)	265(69.9)	102(26.9)	12(3.2)	632(83.4)	126(16.6)	0.15	0.067
<70	569(74.6)	179(23.5)	15(2.0)	1317(86.3)	209(13.7)	598(67.3)	258(29.0)	33(3.7)	1454(81.8)	324(18.2)	**0.0080**	**0.0012**
Gender												
Male	636(75.0)	198(23.2)	15(1.8)	1470(86.6)	228(13.4)	503(69.9)	196(28.0)	15(2.1)	1202(83.9)	226(16.1)	0.16	0.069
Female	324(75.1)	96(22.6)	10(2.3)	744(86.4)	116(13.6)	360(65.7)	164(28.9)	30(5.4)	887(80.1)	221(19.9)	**0.014**	**0.0012**
Diabetes												
Yes	286(78.8)	72(19.8)	5(1.4)	644(88.7)	82(11.3)	84(75.7)	26(23.4)	1(0.9)	194(87.4)	28(12.6)	0.70	0.63
No	674(73.6)	222(24.2)	20(2.2)	1570(85.7)	262(14.3)	773(66.8)	340(29.4)	44(3.8)	1886(81.5)	428(18.5)	**0.0080**	**0.0012**
Hypertension												
Yes	666(74.8)	205(23.0)	20(2.2)	1537(86.3)	245(13.7)	247(70.1)	91(25.9)	14(4.0)	585(83.1)	119(16.9)	0.15	0.068
No	294(75.8)	89(22.9)	5(1.3)	677(87.2)	99(12.8)	616(67.2)	269(29.4)	31(3.4)	1495(81.9)	331(18.1)	**0.0080**	**0.0013**

P_G_: P value of the difference in alleles between the case and control groups; P_A_: P value of the difference in genotype between the case and control groups

^*a*^ adjusted for age, gender, smoking, hypertension, diabetes mellitus and hyperlipidaemia.

P < 0.05 is indicated in bold font.

**Table 6:** A comparison between the baseline characteristics of the ADAMTS7 rs7173743 genotypes and alleles in combined IS patient and control groups.

**Characteristics**	**IS patient group**					**Control group**					***P_G_^a^ value***	***P_A_^a^ value***
	**Genotype n (%)**			**Allele n (%)**		**Genotype n (%)**			**Allele n (%)**			
	**TT**	**TC**	**CC**	**T**	**C**	**TT**	**TC**	**CC**	**T**	**C**		
Age												
≥70	169(32.8)	226(43.8)	121(23.4)	564(54.7)	468(45.3)	101(26.5)	185(48.6)	95(24.9)	387(50.8)	375(49.2)	0.17	0.15
<70	183(24.0)	413(54.1)	167(21.9)	779(51.0)	747(49.0)	186(21.0)	427(48.1)	274(30.9)	799(45.0)	975(55.0)	**5.1×10^-4^**	**0.0016**
Gender												
Male	243(28.6)	410(48.3)	196(23.1)	896(52.8)	802(47.2)	179(25.1)	341(47.8)	194(27.1)	699(48.9)	729(51.1)	0.17	0.054
Female	133(30.9)	206(47.9)	91(21.2)	472(54.9)	388(45.1)	108(19.5)	271(48.9)	175(31.6)	487(44.0)	621(56.0)	**8.8×10^-5^**	**1.4×10^-5^**
Diabetes												
Yes	85(23.4)	192(52.9)	86(23.7)	362(49.9)	364(50.1)	28(25.3)	51(45.9)	32(28.8)	107(48.2)	115(51.8)	0.40	0.70
No	267(29.1)	447(48.8)	202(22.1)	981(53.5)	851(46.5)	259(22.4)	561(48.5)	337(29.1)	1079(46.6)	1235(53.4)	**2.8×10^-4^**	**4.0×10^-5^**
Hypertension												
Yes	246(27.6)	443(49.7)	202(22.7)	935(52.5)	847(47.5)	85(24.2)	177(50.0)	91(25.8)	347(49.2)	359(50.8)	0.38	0.16
No	106(27.3)	196(50.5)	86(22.2)	408(52.6)	368(47.4)	202(22.1)	435(47.5)	278(30.4)	839(45.8)	991(54.2)	**0.011**	**0.0034**

P_G_: P value of the difference in alleles between the case and control groups; P_A_: P value of the difference in genotype between the case and control groups

^*a*^ adjusted for age, gender, smoking, hypertension, diabetes mellitus and hyperlipidaemia.

P < 0.05 is indicated in bold font.

### Association of *ADAMTS7* variants with stroke subtypes

Patients with IS enrolled in our study were subcategorized according to the Trial of Org 10172 in Acute Stroke Treatment (TOAST) classification t to elucidate if *ADAMTS7* variants increased overall risk or were limited to a higher risk of specific stroke subtypes. Stratification by the TOAST classification revealed that, compared to controls, carriers of the rs3825807 G allele (P = 6.0×10^-4^), the rs11634042 T allele (P = 4.4×10^-4^), or the rs7173743 C allele (P = 5.2×10^-4^) had reduced risk of large-artery atherosclerosis (LAA)-subtype stroke (Tables 7–9).

**Table 7:** The relationship between *ADAMTS7* rs3825807 variant and IS stratified by TOAST classification in combined IS patients.

	**ADAMTS7 rs3825807**							
	**Genotype**			**P value^a^**	**Allele**		**P value^a^**	**OR (95% CI)**
	**AA**	**AG**	**GG**		**A**	**G**		
Control (n=1268)	855(67.4)	368(29.0)	45(3.6)		2078(81.9)	458(18.1)		
*Cases*								
LAA (n=812)	607(74.8)	189(23.3)	16(2.0)	**0.0033**	1403(86.4)	221(13.6)	**6.0×10^-4^**	0.72(0.60-0.85)
SAA (n=355)	258(72.7)	88 (24.8)	9(2.5)	0.23	604 (85.1)	106(14.9)	0.069	0.80(0.63- 1.00)
CE (n=46)	36(78.3)	9(19.6)	1(2.2)	0.32	81(88.0)	11(12.0)	0.13	0.62(0.33- 1.17)
UE (n=66)	53(80.3)	12(18.2)	1(1.5)	0.19	118(89.4)	14(10.6)	0.058	0.54(0.31-0.95)

LAA: Large-artery atherosclerosis; SAA: Small-artery atherosclerosis; CE: Cardioembolic; UE: Undetermined aetiology.

**Table 8:** The relationship between *ADAMTS7* rs11634042 variant and IS stratified by TOAST classification in combined IS patients.

	***ADAMTS7* rs11634042**						
	**Genotype**			**P value^a^**	**Allele**		**P value^a^**	**OR (95% CI)**
	**CC**	**CT**	**TT**		**C**	**T**		
Control (n=1268)	863 (68.0)	360 (28.4)	45(3.6)		2086(82.3)	450(17.7)		
*Cases*								
LAA (n=812)	613(75.5)	183(22.5)	16(2.0)	**0.0026**	1409(86.8)	215(13.2)	**4.4×10^-4^**	0.71(0.59-0.84)
SAA (n=355)	259(73.0)	89(25.0)	7(2.0)	0.17	607(85.5)	103(14.5)	0.057	0.79(0.62-0.99)
CE (n=46)	35(76.1)	10(21.7)	1(2.2)	0.64	80(87.0)	12(13.0)	0.25	0.70(0.38 - 1.29)
UE (n=66)	53(80.3)	12(18.2)	1(1.5)	0.17	118(89.4)	14(10.6)	0.057	0.55(0.31 -0.97)

LAA: Large-artery atherosclerosis; SAA: Small-artery atherosclerosis; CE: Cardioembolic; UE: Undetermined aetiology.

**Table 9:** The relationship between *ADAMTS7* rs7173743 variant and IS stratified by TOAST classification in combined IS patients.

	***ADAMTS7* rs7173743**							
	**Genotype**			**P value^a^**	**Allele**		**P value^a^**	**OR (95% CI)**
	**TT**	**TC**	**CC**		**T**	**C**		
Control (n=1268)	287 (22.6)	612 (48.3)	369 (29.1)		1186(46.8)	1350(53.2)		
*Cases*								
LAA (n=812)	224(27.6)	410(50.5)	178(21.9)	**0.0019**	858(52.8)	766(47.2)	**5.2×10^-4^**	0.78(0.69 -0.89)
								
SAA (n=355)	94(26.5)	174(49.0)	87(24.5)	0.19	362 (51.0)	348(49.0)	0.063	0.84(0.72- 1.00)
								
CE (n=46)	14(30.4)	25(54.3)	7 (15.3)	0.18	53(57.6)	39(42.4)	0.063	0.65(0.42- 0.98)
								
UE (n=66)	20(30.3)	30(45.5)	16 (24.2)	0.34	70(53.0)	62(47.0)	0.16	0.78(0.55- 1.10)

LAA: Large-artery atherosclerosis; SAA: Small-artery atherosclerosis; CE: Cardioembolic; UE: Undetermined aetiology.

### Effect of *ADAMTS7* variants on ADAMTS7 expression

**Figure 2:**
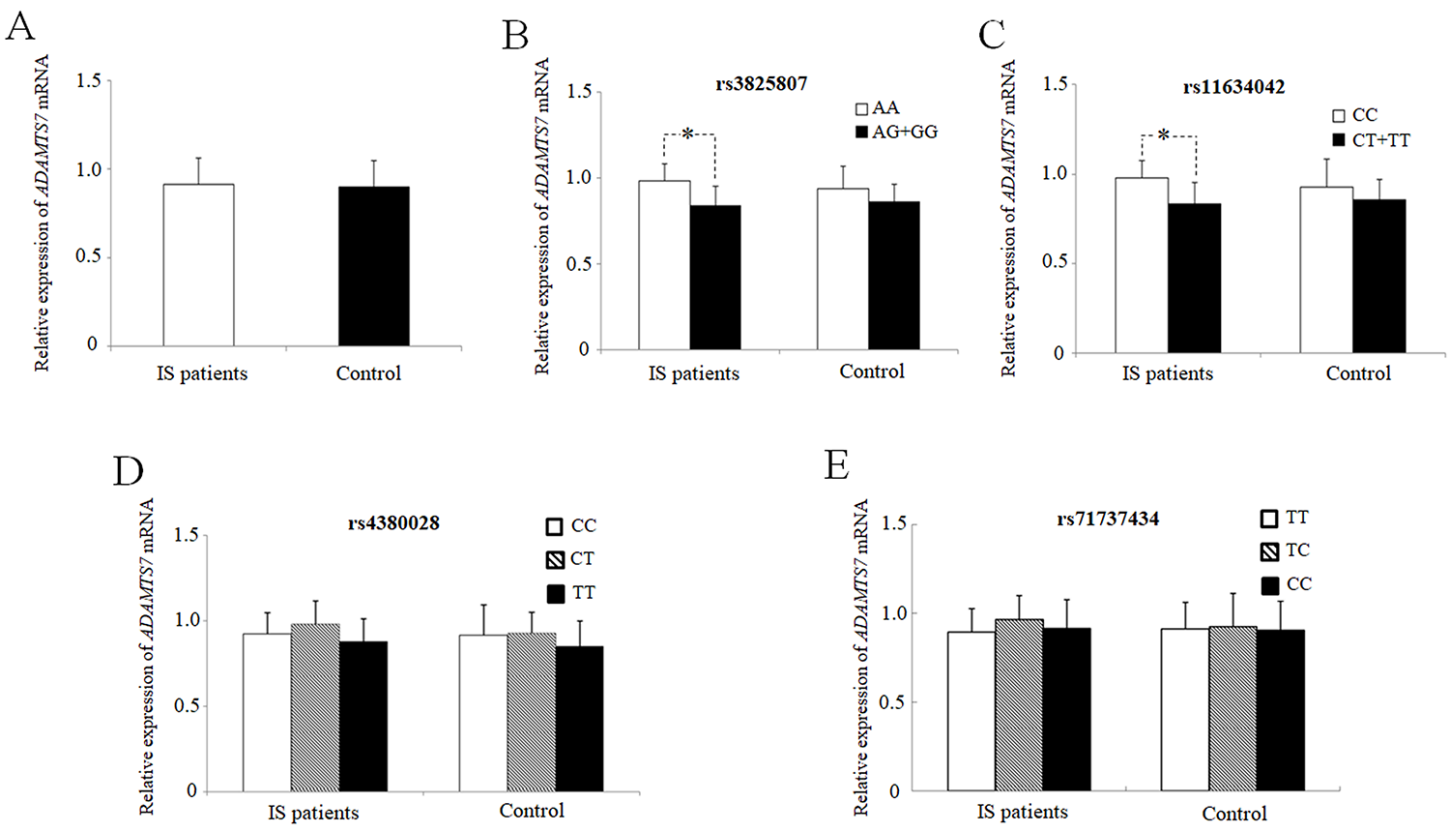
**Mean values ± SD of relative *ADAMTS7* mRNA in PBMCs from IS patients (IS, n = 87) and healthy subjects (controls, n = 50).** the blank box and the black box represent the relative expressions of adamts7 in IS patients and controls, respectively, and the median is indicated by a bar across the box. p=0.63 when comparing relative *adamts7* mRNA levels between IS patients and controls. mean values ± sd of *adamts7* mRNA in pbmcs from IS patients and healthy subjects stratified according to the genotypes and alleles of rs3825807 (**B**) (^*^p = 0.025), rs11634042 (**C**) (^*^p = 0.045), rs4380028 (**D**) and rs7173743 (**E**), respectively. an asterisk indicates p < 0.05.

*ADAMTS7* mRNA expression levels in PBMCs from 87 patients with IS and 70 healthy controls were measured and compared, without significant differences; P = 0.63 (Figure 2A). We also investigated the link between mean *ADAMTS7* mRNA levels in IS patients and the *ADAMTS7* genotype (Figure 2). Significantly reduced *ADAMTS7* mRNA expression was found in IS patients carrying the mutated rs3825807 G allele (P = 0.025; Figure 2B) or the rs11634042 T allele (P = 0.045; Figure 2C). Nevertheless, no such difference was found between carriers of the rs4380028 and rs7173743 alleles and carriers of the major common alleles regarding *ADAMTS7* mRNA expression, in either IS patients or control (Figure 2D and 2E).

### Effect of *ADAMTS7* variants on carotid atherosclerosis

**Figure 3:**
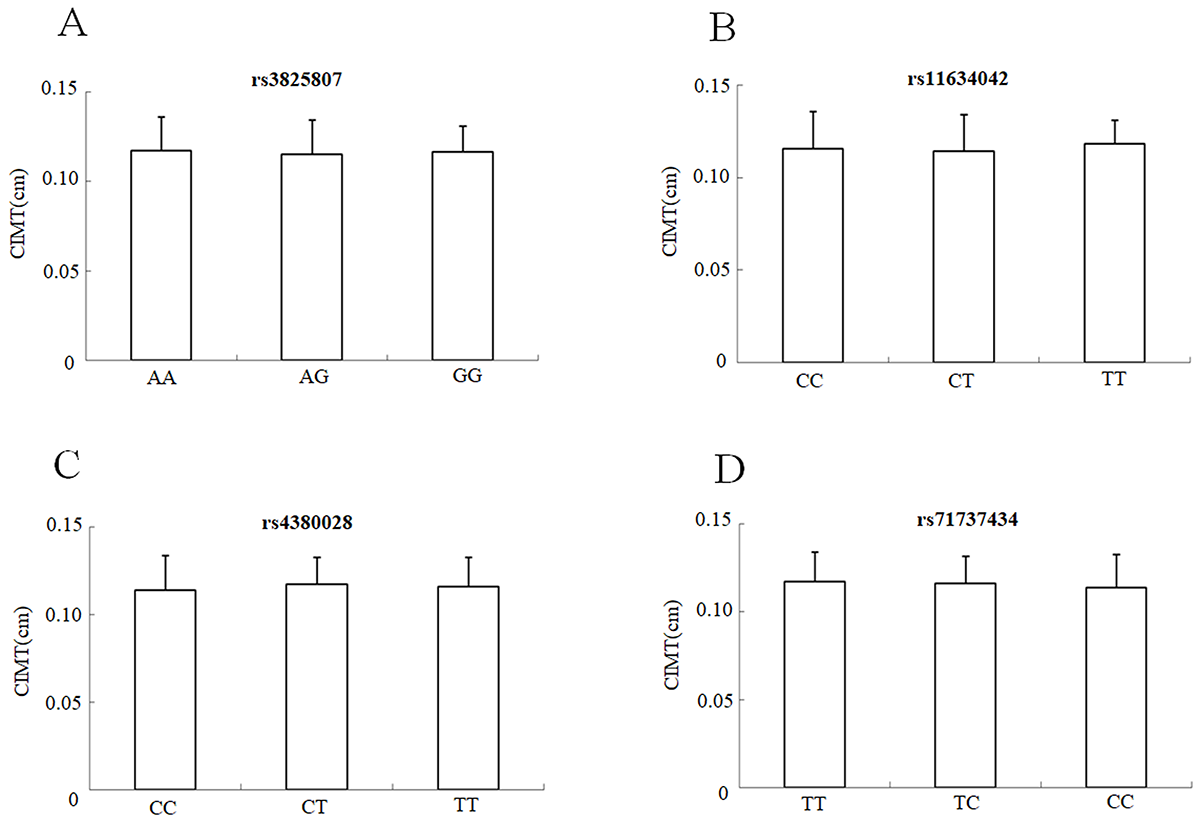
CIMT mean values ± SD of IS patients (n = 264) stratified according to the genotypes and alleles of rs3825807. (**A**), rs11634042 (**B**), rs4380028 (**C**) and rs7173743 (**D**), respectively.

The mean CIMT of the IS patients with the mutated genotypes (rs3825807 GA&AA, rs11634042 CT&TT, rs4380028 CT&TT and rs7173743 CT&TT) and IS patients with the major genotypes (rs3825807AA, rs11634042CC, rs4380028CC andrs7173743TT) was similar, without significant differences; P>0.05 (Figure 3).

## DISCUSSION

Our main finding is that we first demonstrate the rs3825807, rs11634042, and rs7173743 *ADAMTS7* variants to be associated with low IS risk. Assessment of the haplotypes indicated the G-T-T-C and G-T-C-C haplotypes (the rs3825807–rs11634042–rs4380028–rs7173743 variants) were associated with reduced IS risk. Furthermore, the rs3825807 G, rs11634042 T, and rs7173743 C alleles were also linked with a lower risk of LAA-subtype stroke after stratification analysis. In addition, the rs3825807 and rs11634042 variants appeared to modulate ADAMTS7 expression.

Research has increasingly supported the role of ADAMTS7 as a promoter of neointimal proliferation, plaque development, and plaque calcification, as the underlying pathology for vast majority of IS patients. Overexpression of ADAMTS7 through adenoviral infection enhanced VSMC proliferation and mobility *in vitro* and increased neointimal formation in rats. In contrast, ADAMTS7 downregulation mediated by small interfering RNA (siRNA) had the opposite effect [7]. In human coronary and carotid atherosclerotic plaques, ADAMTS7 is most prominent in the proximity of the intima-media border and the fibrous cap [15]. The *ADAMTS7* rs3825807 G allele has been noted to affect ADAMTS7 maturation, while COMP cleavage and VSMC migration has been linked with subclinical atherosclerosis. ADAMTS7 upregulation mediated by miR-29a/b repression facilitates vascular calcification both *in vitro* and *in vivo* [18]. Although ADAMTS7 is widely recognized to intervene in the pathophysiology of atherosclerosis, the *ADAMTS7* alleles specifically related to IS have yet to be elucidated.

Various *ADAMTS7* SNPs have been linked with CAD in isolated GWA studies [11–13]. To date, rs3825807 is the most widely studied functional SNP in this group and has been reported to suppress ADAMTS7 maturation, COMP cleavage, and VSMC migration [15]. Likewise, the *ADAMTS7* rs3825807 GG genotype appears to have an inverse correlation with atherosclerosis, reflected in lower frequency and magnitude of carotid atherosclerosis [15]. Also, in patients with CAD, the mutant G allele of the *ADAMTS7* rs3825807 variant appears to improve cardiovascular survival [16]; as well as reduced severity of the CAD phenotype [17]. In a Chinese cohort, You *et al.* found *ADAMTS7* rs3825807 is related to CAD risk and severity [19]. This study is the first to demonstrate lower IS risk in subjects with the mutant alleles at the rs3825807, rs11634042and rs7173743 locus of *ADAMTS7,* as well as the G–T–T–C and G-T-C-C haplotypes, which corresponds to the rs3825807–rs11634042–rs4380028–rs7173743 variants.

Our findings suggested the variant G allele of *ADAMTS7* rs3825807 may protect against stroke, in harmony with GWA studies which have linked the G allele of with lower CAD risk. Our study also reported for the first time that the rs7173743 variant was associated with decreased risk of IS; this result is in agreement with the findings associating this variant with coronary artery calcium contents in Hispanics [20]. Although GWA studies have also identified *ADAMTS7* rs4380028 as a novel susceptibility locus for CAD, a case-control study of the Japanese population failed to replicate the association between this variant and coronary atherosclerosis [21]. Consistent with this report, we did not find any significant associations between the *ADAMTS7* rs4380028 variant and IS. Nevertheless, further studies are needed to fully explain the associations between this gene and the incidence of IS in different populations.

The rs3825807 G/G genotype in the *ADAMTS7* locus, has been linked with reduced prevalence and severity of atherosclerosis, yet does not appear to reduce the expression of ADAMTS7 but its maturation and activity, resulting in reduced COMP cleavage and attenuated VSMC migration [15]. Another recent study by Bayoglu *et al.* demonstrated greater ADAMTS7 expression in patients with peripheral artery disease and the AA genotype of rs3825807 [22]. In our present study, ADAMTS7 expression was diminished in IS patients with the G allele of rs3825807 and the T allele of rs11634042. The rs3825807 Ser214Pro variant may impact ADAMTS7 expression due to the change of a polar amino acid (serine) for a non-polar one (proline) in the protein’s prodomain. This assumption is also supported by reports associating the protective T allele of *ADAMTS7* rs7178051—which is modestly linked to disequilibrium with rs3825807 (LD=0.52)—with lower ADAMTS7 expression in human aortic endothelial cells and lymphoblastoid cells [23]. Unexpectedly, our study found *ADAMTS7* expression to be similar in both IS patients and controls. We speculate that this lack of a difference is not because ADAMTS7 role in IS, rather, it is likely compromised by other unknown factors, such as other genetic variants of *ADAMTS7* or interactions with other environmental factors.

Because dysregulated degradation of the ECM in vascular walls is a central phenomenon in the pathophysiology of atherosclerosis in carotid arteries, ADAMTS7 might be implicated in the early stages of vascular remodelling, thus promoting thickening of the CIMT. However, Hispanics enrolled in the MESA (Multi-Ethnic Study of Atherosclerosis) cohort, were found to have various *ADAMTS7* SNPs related to coronary artery calcification, without impact on CIMT [20]. Similarly, a very recent study performed by Chan *et al.* found no link between whole intima or media thickness with the G allele of the *ADAMTS7* rs3825807 variant [17]. Consistent with these reports, we also found no association between *ADAMTS7* variants and CIMT in IS patients*,* thus offering limited support to the notion *ADAMTS7* variants are direct risk factors for CIMT, which is most prominently in earlier disease stages. The lack of association appears to stand despite the implications of ADAMTS7 in vascular remodelling and thickening, as well as atherosclerotic plaque development. Further studies with broader samples should re-examine the effect of *ADAMTS7* variants on CIMT.

Given the limitations, caution is advised when interpreting results from this study. First, ADAMTS7 levels were compared with the genotypes only in a small number of patients and controls (87 IS patients vs. 70 healthy controls), which may have caused a type II error. Second, information, selection, and confounding bias cannot be excluded with total certainty. Controls were judged as IS-free solely through their medical history, without confirmation with neuroimaging. The lack of imaging in the control group may implicate a confounder by the inclusion of controls with a history of silent stroke, thereby altering statistical analyses. Other confounding variables include risk factors such as age, gender, and the presence of comorbidities. Third, additional functional variants may also modulate ADAMTS7 expression and intervene in IS. These combined effects require further exploration to enhance the prediction of IS. Meanwhile, modulating these factors decrease the occurrence, severity, and outcomes of IS patients. Fourth, circulating COMP levels were not examined, meaning the impact of *ADAMTS7* variants on COMP expression in IS patients also went unassessed. Indeed, before drawing definitive conclusions from our findings, further confirmation is necessary for large independent samples, including subjects with varying ethnicities. This would highlight their usefulness for estimating IS risk in different individuals.

This study is the first to show the *ADAMTS7* rs3825807, rs11634042 and rs7173743 variants confer a lower risk of IS. Furthermore, the *ADAMTS7* rs3825807 and rs11634042 variants may modulate the genetic predisposition to IS by reducing ADAMTS7 expression. This new piece of knowledge regarding ADAMTS7 may be clinically significant for the prevention and personalized therapy of IS.

## MATERIALS AND METHODS

### Subject recruitment and sample collection

This two-stage case-control study recruited 1279 IS patients and 1268 controls. The initial cohort comprises 615 individuals with IS (408 men, 207 women) recruited by the Department of Neurology at the Affiliated Hospital of Guangdong Medical University between 2015 and 2017. To confirm the significant statistics observed in the initial cohort, a replication cohort included 664 IS patients and 653 healthy individuals were consecutively recruited from the First Affiliated Hospital of Harbin Medical University from 2015 to 2018. All human subjects in initial cohort and replication cohort were genetically unrelated Han Chinese adults from Guangdong and Heilongjiang Province. The diagnosis of IS was established by the assessment of clinical symptoms and physical examination; as well as neuroimaging. Patients were categorized using the TOAST system [24]. On the other hand, we excluded subjects with previously established transient ischaemic attacks, coronary artery disease, haemorrhagic stroke, subarachnoid hemorrhage, chronic infections, cancer, and hematologic, immunologic and systemic inflammatory disorders. We also excluded one patient who had previously suffered an IS.

The initial control group included 615 age-matched and ethnicity-matched subjects (311 men, 304 women) who consulted at the Health Examination Center of the Affiliated Hospital of Guangdong Medical University in parallel to the IS patients regarding time. The replication control subjects randomly selected 653 sex- and age-matched healthy individuals (403 men, 250 women) from the same geographical area (Central Harbin) within the same period. The control subjects with recent myocardial infarction or cerebrovascular disease were excluded. The replication cohort followed the same inclusion and exclusion criteria as the initial one.

All subjects provided written, informed consent. All procedures in this study comply with the stipulations of the Declaration of Helsinki. Likewise, the Ethics Committee of the Affiliated Hospital of Guangdong Medical University and the First Affiliated Hospital of Harbin Medical University approved all protocols.

### SNP selection and genotyping

The four *ADAMTS7* SNPs (rs3825807, rs11634042, rs4380028, rs7173743) were chosen with guidance from prior research [12, 20, 21, 25]. Circulating leukocytes from each subject were processed with a TIANamp Blood DNA kit (Tiangen Biotech, Beijing, China) according to the manufacturer’s instructions for the extraction of genomic DNA. DNA purity and concentration were evaluated with a DNA spectrophotometer (ND-1000, NanoDrop, Wilmington USA). The SNaPshot Multiplex Kit (Applied Biosystems Co., Ltd., Foster City, CA, USA) was implemented for genotyping the *ADAMTS7* SNPs, utilizing the primers shown in Supplementary Table 1 proceeding as described in a previous publication [26].

### Extraction of the RNA and Real-Time PCR

Lymphoprep^TM^ (Axis-Shield PoCAS, Oslo, Norway) was used for isolation of peripheral blood mononuclear cells (PBMC) *via* centrifugation with a density gradient, as described in a previous report by our group [26]. The RNAprep pure Blood Kit (TianGen Biotech, Beijing, China) was used following the manufacturer’s indications to obtain total cellular RNA from the PBMC. The silica membrane was subjected to digestion with RNase-free DNase I to mediate the removal of genomic DNA residues. The cDNA Synthesis Kit RevertAid (Thermo) was used as per the manufacturer’s instructions to convert total RNA to cDNA. The cDNA obtained (10 ng) were used as a template to determine the quantity of *ADAMTS7* and *GAPDH* with quantitative real-time PCR, implementing the SYBR green method as previously described [26]. *ADAMTS7* and *GAPDH* mRNA was quantified in three independent measures. Then, the relative mRNA levels were estimated with the 2^-ΔΔCt^ method [27] and normalized by the housekeeping gene glyceraldehyde-3-phosphate dehydrogenase (GAPDH). This method directly uses the threshold cycles generated by the qPCR system, rendering it particularly convenient and efficient for the assessment of the relative expression of both target and control genes among different samples. The following RT-PCR primers were used in the assay: *ADAMTS7* sense primer, GGTCGGTCAGCAAAGAGAAG; *ADAMTS7* anti-sense primer, CCATGTTCATGATGGTCAGC; *GAPDH* sense primer, GAAGGGCTCATGACCACAGTCCAT; and *GAPDH* anti-sense primer, TCATTGTCGTACCAGGAAATGAGCTT. Relative expression was calculated in each sample based on technical triplicate results. Products from amplification were validated *via* melting curve analysis.

### Ultrasound assessment

A 7.5- to 10.0-MHz linear array ultrasonographic transducer (P700SE; Phillips Medical System) was implemented in the B-mode for assessing carotid intima-media thickness (CIMT) in both the right and left arteries, and in the near and far walls. Optimized images depicting CIMT in both the left and right arteries were chosen and paused at the end of the diastole. Maximum CIMT-i and CIMT-c were calculated by averaging the maximum values of the near and far walls in both the right and left sides, in an area free of atherosclerotic plaque, as previously described [26].

### Statistical evaluation

SPSS v19.0 (IBM, Armonk, NY, USA) and GraphPad Prism v4.0 (GraphPad Software, Inc., San Diego, CA, USA) were utilized for statistical analyses. The Hardy–Weinberg equilibrium (HWE) of the SNPs was evaluated with specialized software for this purpose. Haplotype examination was performed with the Haploview v4.2. Continuous data are displayed as means ± standard deviation (SD), and the median or percentage for categorical variables. Comparisons among groups were conducted with the Chi-squared (χ2) test and Student’s t-test. Data with normal distribution were assessed with Student’s t-test; the Mann-Whitney U test was used otherwise. Fisher’s exact test or the χ2 test were implemented for comparing the frequencies of the *ADAMTS7* allele and specific genotypes between patients with IS and controls. Correlations between *ADAMTS7* genotypes and IS were analyzed *via* generation of odds ratios (OR) with 95% confidence intervals (CI). Multiple linear regression analysis of the associations between *ADAMTS7* variants and CIMT was conducted by constructing relevant models with SAS v6.12 (SAS Institute Inc., Cary, NC, USA). These were adjusted for age and sex, as well as comorbidities such as diabetes mellitus, hypertension, and dyslipidemia. Multiple comparisons with control type 1 error underwent correction with the Bonferroni method. Statistical significance was set at *p*<0.05.

## Supplementary Material

Supplementary Table 1
