# Supplementary material for: Genetic variants of ADAMTS7 confer risk for ischaemic stroke in the Chinese population
Source: Aging (Albany NY). 2019 Aug 28;11(16):6569–83. doi: 10.18632/aging.102211 (PMC6738416; doi:10.18632/aging.102211)
Supplement: Supplementary Table 1 [file aging-11-102211-s001.pdf]

## SUPPLEMENTARY TABLE

**Supplementary Table 1. Primers used in genotyping of *ADAMTS7* variants.**

| Gene variants | Direction | Primers |
|---|---|---|
| **rs11634042** | forward | 5′-ACACGACGCTCTTCCGATCTCATCCTACCATGCACACTGCATA-3′ |
| | reverse | 5′-TTCCTTGGCACCCGAGAATTCCATGTGCCACTATACCCAGCTATTATCA-3′ |
| **rs3825807** | forward | 5′-ACACGACGCTCTTCCGATCTTGGTCAGCACATAGCTCTCAAC-3′ |
| | reverse | 5′-TTCCTTGGCACCCGAGAATTCCACAGAGAGAGCTCACTGAGGAGA-3′ |
| **rs4380028** | forward | 5′-ACACGACGCTCTTCCGATCTTGGGCAGTGAGAATGATGATTTGA-3′ |
| | reverse | 5′-TTCCTTGGCACCCGAGAATTCCAAATTTGGAGAGGCAGTGATATGGAG-3′ |
| **rs7173743** | forward | 5’-ACACGACGCTCTTCCGATCTAAGAGCTGGTATTGTTTTGGACCA-3′ |
| | reverse | 5′-TTCCTTGGCACCCGAGAATTCCAGGCTGAGACAGTTTCGAGGATTAG-3′ |
